# Effect of Aging on the Morphofunctional Characteristics of Oral Cavity Mesenchymal Stromal Cells: A Scoping Review

**DOI:** 10.3390/biomedicines13112776

**Published:** 2025-11-13

**Authors:** Josefa Alarcón-Apablaza, Luis A. Salazar, Pía Loren, Constanza Martínez-Cardozo, Ramón Fuentes

**Affiliations:** 1 Research Centre in Dental Sciences (CICO-UFRO), Dental School, Universidad de La Frontera, Temuco 4780000, Chile; ramon.fuentes@ufrontera.cl; 2 Doctoral Program in Morphological Sciences, Faculty of Medicine, Universidad de La Frontera, Temuco 4780000, Chile; 3 Center of Molecular Biology and Pharmacogenetics, Department of Basic Sciences, Faculty of Medicine, Universidad de La Frontera, Temuco 4780000, Chile; luis.salazar@ufrontera.cl; 4 Laboratorio de Investigación en Salud de Precisión, Departamento de Procesos Diagnósticos y Evaluación, Facultad de Ciencias de la Salud, Universidad Católica de Temuco, Temuco 4780000, Chile; ploren@uct.cl; 5 Department of Biomedical Sciences, Ethics, Research and Education, Faculty of Dentistry, Center for Biomedical Research and Innovation (CIIB), Universidad de Los Andes, Santiago 7620086, Chile; cemartinezc@uandes.cl; 6 Department of Integral Adult Dentistry, Dental School, Universidad de La Frontera, Temuco 4780000, Chile

**Keywords:** MSCs, mesenchymal stem cells, senescence, oral cavity

## Abstract

Over the past decade, interest has grown in understanding the morphofunctional changes that mesenchymal stem cells (MSCs) undergo due to age-associated senescence—a process particularly relevant given that adults and elderly individuals are the primary candidates for regenerative therapies. This study addresses this knowledge gap by systematically analyzing the influence of age-related senescence on the morphofunctional properties of MSCs derived from the oral cavity. A scoping review was conducted following the PRISMA-ScR guidelines. The databases searched were MEDLINE, SCOPUS, and Web of Science. In vitro studies were included if their primary objective was to investigate oral cavity mesenchymal stromal cells and age-related senescence. A total of 455 studies were identified, of which 17 were selected. Studies on MSCs from the oral cavity have shown that age-related senescence, starting around 35 years, reduces proliferation, viability, clonogenic capacity, and differentiation potential—particularly toward osteogenic and chondrogenic lineages—with higher values observed in younger individuals. However, MSC surface markers remain stably expressed and show no association with aging. Some studies also report no significant differences in proliferation rate or cell doubling time at early passages, and MSCs retain some plasticity at these stages. Despite age-related limitations, oral MSCs from elderly donors remain a promising therapeutic source, especially at early in vitro passages. Further research is needed to explore innovative strategies to enhance the regenerative potential of oral MSCs from older donors.

## 1. Introduction

Mesenchymal stromal cells (MSCs) are non-hematopoietic cells derived from the mesoderm, with an intrinsic capacity for self-renewal. They have potent regenerative properties and the potential for multilineage differentiation [1,2,3,4,5]. Furthermore, MSCs can evade the immune system by being immunomodulatory, allowing them to be used in therapeutic functions [6]. Due to these characteristics, MSCs have generated growing interest in a wide variety of biomedical disciplines. MSCs were initially discovered in bone marrow (BM) in 1976. BM is considered the major source of mesenchymal stromal and progenitor cells for experimental and clinical applications [1]. Although bone marrow represents the most widely studied source of MSCs, it has some limitations. The main drawback of BM is the low MSC yield, ranging from 0.001 to 0.01% of the total cell population, allowing the isolation of only 60–600 cells per ml of BM aspirate [7,8]. Furthermore, although BM-MSCs have a self-renewal capacity and differentiation potential, they undergo replicative senescence [9]. For this reason, the study of alternative sources of MSC is particularly important.

Several studies have identified the presence of neural crest-derived stem cells (NCSCs) in various adult craniofacial tissues [2,5,6,10]. Due to their embryonic origin and minimally invasive extraction, intraoral tissues are considered a promising source of stem cells for tissue engineering applications, especially in regenerative dentistry [11,12,13]. These cells have demonstrated a remarkable differentiation capacity in various cell lineages, such as osteoblasts, chondrocytes, and adipocytes [14,15]. Additionally, they are easy to isolate and exhibit a higher proliferation rate than MSCs from bone marrow, even without the need for growth factors. This allows large numbers of MSCs to be obtained from a small tissue sample in short-term primary cultures [16,17].

A notable advantage of oral cavity MSCs is their ease of autologous collection through minimally invasive, routine dental procedures (e.g., tooth extractions or periodontal surgeries), thereby eliminating concerns regarding donor site morbidity, immune rejection, or the need for immunosuppressive therapy [18,19]. Successful results have been demonstrated with oral cavity MSC autografts in young subjects [11]; however, adults are the primary population requiring regenerative interventions, and the effects of aging on mesenchymal stem cell function represent an important consideration for the success of such therapies.

Aging is a multifactorial biological process characterized by a progressive functional decline that affects different organ systems in a heterogeneous manner in mammals, reducing the capacity to maintain homeostasis and increasing susceptibility to diseases and tissue dysfunction [20]. In contrast, cellular senescence is defined as a stable state of cell-cycle arrest induced by various types of stress, including telomere shortening, DNA damage, epigenetic dysregulation, and mitochondrial dysfunction. Although senescence plays essential physiological roles during development and contributes to tissue homeostasis, its chronic accumulation is associated with multiple aspects of aging, including loss of regenerative function and the promotion of a proinflammatory microenvironment through the senescence-associated secretory phenotype (SASP) [21,22]. Therefore, cellular senescence is considered a central mechanism contributing to biological aging and the onset of age-related diseases.

Studies on the performance of MSC isolation from elderly individuals show significant changes in morphofunctional characteristics due to cellular senescence, which may limit their autologous use in adult subjects [23,24]. Senescence is a cellular response characterized by a stable cell cycle arrest that limits the proliferative potential of cells. To date, four types of senescence have been distinguished: (1) replicative senescence (RS), which occurs during cell passage subculture; (2) oncogene-induced senescence (OIS); (3) stress-induced premature senescence (SIPS); and (4) developmental senescence [25,26,27,28]. We present these categories to highlight how age-related senescence differs from the other types.

Age-related senescence results from the cumulative activation of multiple cellular stress pathways in vivo over time and is manifested through both morphological features—such as increased cell size and granularity—and functional alterations, including reduced proliferation, growth, migration, immunomodulation, and differentiation capacity. Replicative senescence in MSCs, which has been extensively described in the literature, is associated with distinctive morphofunctional alterations, including increased granularity, enlarged and flattened morphology, telomere attrition, epigenetic remodeling, impaired differentiation potential, elevated SA-β-gal activity, altered autophagy, increased ROS generation, G1 cell cycle arrest, and upregulation of the p53 and p21 pathways [23,29,30,31]. These changes lead to reduced plasticity and proliferative potential, ultimately compromising regenerative capacity and limiting therapeutic applications [32,33]. However, despite the extensive characterization of replicative senescence in vitro, the specific features of aging-induced senescence in MSCs derived from the oral cavity remain less clearly defined. Current evidence suggests that in vivo aging may trigger additional senescence mechanisms influenced by microenvironmental and tissue-specific stressors. Therefore, in this review, studies focusing on replicative senescence were excluded in order to specifically address the effects of chronological donor age without the confounding influence of in vitro passaging.

Understanding the morphofunctional changes in MSCs resulting from age-related senescence has gained importance in the last decade, since adults and elderly subjects are the ones most in need of regenerative treatments. However, knowledge about how age-related senescence affects MSCs in the oral cavity is limited and often contradictory. Therefore, how the donor’s age modulates the morphofunctional characteristics of MSCs is unclear. This study aims to explore the effect of age-related senescence on the morphofunctional characteristics of mesenchymal stem cells (MSCs) extracted from the oral cavity. Understanding how age influences these properties is essential to overcoming the current limitations of autologous tissue engineering in adult patients.

## 2. Materials and Methods

### 2.1. Systematic Literature Search

A scoping review was performed on MSCs from the oral cavity and age-induced cellular senescence. Our scoping review was performed according to the Preferred Reporting Items for Systematic Reviews and Meta-Analyses extension for Scoping Reviews (PRISMA-ScR) guidelines [34].

An electronic search was carried out in three digital databases (MEDLINE, SCOPUS, and Web of Science). The search terms selected were: “Stem Cells”, “Mesen-chymal Stem Cells”, “Mesenchymal Stromal Cells”, “MSCs”, “Multipotent Stromal Cells”, “Mesenchymal Progenitor Cells”, “Progenitor Cells”, “Cells Mother”, “Stem Cell Mes-enchymal”, “Adult Stem Cell”, “Oral Cavity”, “Cavity Oral”, “Cavitas Oris”, “Maxil-lofacial”, “Stomatognathic”, “Mouth”, “Senescence”, “Longevidade”, “Age Longevity”, “Aging”, “Cellular Senescence”, “Cellular Senescence”, “Senescence-Associated Secretory Phenotype”. The keywords were combined with Boolean terms OR and AND. The search was performed between December 2024 and March 2025. The bibliographies of systematic reviews were also screened for any additional studies that were possibly fit for inclusion.

The full search query in the listed databases looked like this: “((((((((((((((((((((((((((((((“Stem cells “[Title/Abstract]) OR (“mesenchymal stromal cells”[Title/Abstract])) OR (“MSC”[Title/Abstract])) OR (“MSCs”[Title/Abstract])) OR (“Mesenchymal stem/stromal cells”[Title/Abstract])) OR (“multipotent stromal cells”[Title/Abstract])) OR (“mesenchymal progenitor cells”[Title/Abstract])) OR (“Progenitor Cells”[Title/Abstract])) OR (“Cell, Progenitor”[Title/Abstract])) OR (“Mother Cell”[Title/Abstract])) OR (“Cells, Stem”[Title/Abstract])) OR (“Cell, Stem”[Title/Abstract])) OR (“Mother Cells”[Title/Abstract])) OR (“Cell, Mother”[Title/Abstract])) OR (“Cells, Mother”[Title/Abstract])) OR (“Stem Cell, Mesenchymal”[Title/Abstract])) OR (“Mesenchymal Stem Cell”[Title/Abstract])) OR (“Mesenchymal Stromal Cell”[Title/Abstract])) OR (“Stromal Cell, Mesenchymal”[Title/Abstract])) OR (“Stromal Cells, Mesenchymal”[Title/Abstract])) OR (“Mesenchymal Progenitor Cell”[Title/Abstract])) OR (“Adult Stem Cell”[Title/Abstract])) OR (“Somatic Stem Cell”[Title/Abstract])) OR (“Stem Cell, Somatic”[Title/Abstract])) OR (“Stem Cells”[Mesh])) OR (“Mesenchymal Stem Cells”[Mesh])) OR (“Adult Stem Cells”[Mesh])) OR (“Multipotent Stem Cells”[Mesh])) OR (“Stromal Cells “[Mesh])) AND ((((((“Oral Cavity”[Title/Abstract]) OR (“Cavity, Oral”[Title/Abstract])) OR (“Cavitas Oris”[Title/Abstract])) OR (“maxillofacial”[Title/Abstract])) OR (“stomatognathic”[Title/Abstract])) OR (“Mouth”[Mesh]))) AND ((((((((Senescence[Title/Abstract]) OR (longevidade [Title/Abstract])) OR (Age [Title/Abstract])) OR (“Longevity”[Mesh])) OR (“Aging”[Mesh])) OR (“Cellular Senescence”[Mesh])) OR (“Cellular Senescence”[Mesh])) OR (“Senescence-Associated Secretory Phenotype”[Mesh]))”

The same search equation was adapted for the other search engines. The summary of the factors considered in this review is indicated in Table 1.

### 2.2. Eligible Criteria

In vitro studies were included where the general objective was to study oral cavity mesenchymal stromal cells and senescence. Potentially eligible articles were screened based on the inclusion criteria: studies in English, Spanish, and Portuguese, full text with no publication date limit, and studies that described the effect of aging-induced cellular senescence throughout the life cycle on mesenchymal stromal cells from the oral cavity. Animal studies, studies using stem cells from a site other than the oral cavity, and articles evaluating replicative senescence were excluded.

### 2.3. Data Extraction

Two independent reviewers analyzed articles obtained in the systematic search process by reviewing the titles and abstracts. The articles that met the eligibility criteria were examined in full text to confirm their relevance. In cases of disagreement between the two reviewers, a third reviewer was invited to help resolve the differences in opinion. From the full-text articles that made up the final selection, relevant aspects of age-related senescence and MSC from the oral cavity were compiled. The information collected for Table 1 was: author, year of publication, source of origin of the mesenchymal stromal cells, mesenchymal stromal cells, age of the subjects, study groups, culture methodology, cell maintenance, and experimental passages. The information compiled provides the results of the morphofunctional analyses performed in the studies, including aspects such as cell morphology, MSC markers, cellular senescence, colony formation assays, cell proliferation, PCR, cell migration, immunomodulation, and cell differentiation capacity. The table used in data extraction was designed by the authors of this review to obtain data relevant to the subject studied.

## 3. Results

### 3.1. Study Selection

**Figure 1:**
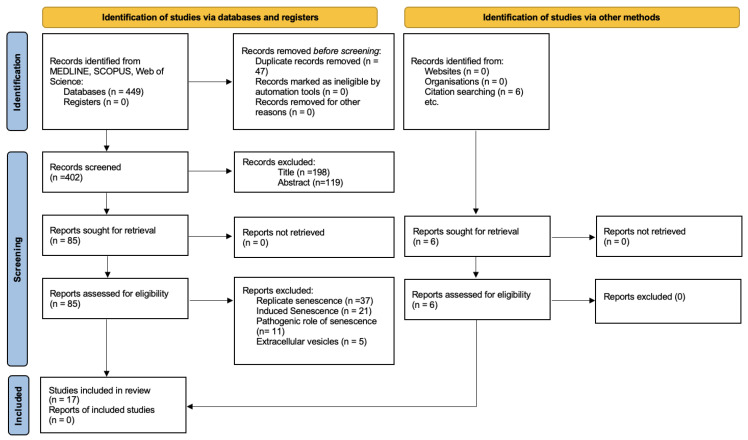
Flow chart for study selection.

The article search and selection process is summarized in Figure 1. A total of 449 articles were identified through database searches, and 6 additional articles were found through manual searching. Of these, 47 were duplicates.

After the initial screening by title, 198 articles were excluded: 83 studied stem cells not derived from the oral cavity, 62 were animal studies, 29 were systematic reviews, 13 focused on methods to enhance cellular senescence, and 11 described anti-aging treatments based on stem cells.

Subsequently, 119 articles were excluded based on abstract screening: 44 studied non-oral stem cells, 28 described anti-aging treatments using stem cells, 19 analyzed replicative senescence under long-term culture conditions, 16 were unrelated to the review topic, and 12 were systematic reviews.

After full-text review of 91 articles (85 obtained by database and 6 articles objectified by other methods), 74 were excluded: 37 focused on replicative senescence, 21 induced senescence through various methods, 11 evaluated cellular senescence as a mechanism for diseases such as primary Sjögren’s syndrome, and 5 analyzed extracellular vesicles. Ultimately, 17 in vitro studies were included in this review [13,35,36,37,38,39,40,41,42,43,44,45,46,47,48,49,50].

### 3.2. Characteristics of the Selected Studies

This article analyzes the effects of age-related senescence on mesenchymal stromal cells (MSCs) derived from the oral cavity. Data were extracted from in vitro studies and are summarized in Table 2 and Table 3, which detail the relevant information from the included studies [13,35,36,37,38,39,40,41,42,43,44,45,46,47,48,49,50].

The methodology for cell culture and maintenance was similar across the analyzed studies. The primary isolation techniques included enzymatic digestion with collagenase and dispase [13,36,37,38,39,40,41,43,44,45,46,48,49], and explant culture methods [35,42,47,50].

Two main protocols were described for enzymatic digestion. In one approach, following enzymatic digestion, the tissue was washed, resuspended in culture medium, and directly plated onto culture dishes [13,38,43,45]. In the other, the cell suspension was filtered through 70 μm cell strainers after washing, in order to obtain a more purified MSC population [36,40,41,44,48,49].

Cell culture and maintenance were performed using complete culture media, generally consisting of Alpha minimum essential medium (α-MEM) [13,37,39,40,41,43,45,47,49,50] or Dulbecco’s modified Eagle medium (DMEM) [35,38,46,48], supplemented with 10% fetal bovine serum (FBS) and 1% penicillin/streptomycin. All cultures were incubated at 37 °C in a humidified atmosphere containing 5% CO_2_.

In this review, “early passages” refer to passages around P5, “extended passages” correspond to P10, and “late passages” indicate P15. Both early and extended passages were used in the included studies. Early passages, specifically between P3 and P5, were most commonly employed to minimize the influence of replicative senescence on experimental outcomes. These details are summarized in Table 2 [37,38,44,47,48,49].

## 4. Discussion

MSCs derived from the oral cavity hold great promise for clinical applications due to their ease of collection, high proliferative capacity, differentiation potential, and suitability for autologous use [11]. These attributes substantially reduce the risk of immune rejection and the need for immunosuppression [18,19]. However, recent studies have demonstrated that MSCs (DPSCs, PDLSCs, GMSCs, and PCs) from elderly individuals exhibit significant alterations in their morphofunctional properties, largely attributable to age-related senescence processes [23,24]. Cellular senescence plays a pivotal role in the functional decline of MSCs derived from the oral cavity, as aging leads various cells—including mesenchymal cells—to age-related senescence traits that adversely impact their regenerative potential [51]. In this context, this study aims to analyze the impact of aging throughout the life cycle on the morphofunctional characteristics of MSCs derived from oral cavity tissues.

Only in the last decade have the effects of age-related senescence on MSCs derived from the oral cavity begun to be described [25,35,36,37,38,39,40,41,42,43,44,45,46,47,48,49,50]. These effects have been primarily studied in cells from the dental pulp and periodontal ligament. However, in recent years, they have also been reported in MSCs from the gingiva [13] and periosteum [41].

### 4.1. Cellular Senescence

Studies have shown that MSCs, isolated from older individuals exhibit reduced proliferative capacity than those from younger individuals, a phenomenon commonly referred to as age-related senescence. MSCs derived from the oral cavity as DPSCs, PDLSCs, and GMSCs from older donors display typical characteristics of senescent cells, including a flattened and enlarged morphology, an increased proportion of senescence-associated β-galactosidase (SA-β-gal)-positive cells, and a decreased proliferation rate [13,26,43,45,46].

SA-β-gal is the most widely accepted marker for assessing cellular senescence, since its initial description by Dimri et al. [52,53]. This lysosomal hydrolase enzyme catalyzes the conversion of β-galactosides into monosaccharides in an acidic environment (pH ~4), an activity present in most mammalian cells regardless of their age [54]. However, its detection at pH 6 makes it possible to differentiate between young and senescent cells, which is why it has been established as the standard for identifying senescent cells [53]. Cellular senescence is also associated with telomerase downregulation and progressive telomere shortening, both considered key markers of senescence [55].

The reviewed studies indicate that SA-β-gal expression increases with donor age [43,45,46], while a decrease in telomere length is observed in DPSCs and PDLSCs from older subjects [46,48]. Another hallmark of the senescent state is irreversible cell cycle arrest. In this process, the activation of the cyclin-dependent kinase (CDK) inhibitor p16^INK4A plays a fundamental role, as its expression blocks cell cycle progression by antagonizing CDKs [46]. In in vitro studies, p16^INK4A^-positive cells have also been shown to exhibit SA-β-gal activity, reinforcing its role as a key marker of senescence [46].

### 4.2. Secretome

MSCs from the oral cavity exert many of their regenerative effects through paracrine signaling, commonly referred to as the secretome [56]. The secretome encompasses all bioactive factors released by MSCs into the extracellular space, including both soluble molecules—such as cytokines, chemokines, growth factors, and immunomodulatory proteins—and vesicular components, including microvesicles and exosomes that mediate intercellular communication via proteins, mRNAs, miRNAs, and lipids [56,57,58]. Experimental evidence suggests that the therapeutic effects of MSC grafts in damaged tissues are largely mediated by these secreted factors rather than direct cellular engraftment [56,59]. The oral MSC secretome has been shown to stimulate cell proliferation, modulate inflammation, and support tissue regeneration under both physiological and pathological conditions, although its composition can vary depending on the microenvironment [58].

Aging and cellular senescence markedly influence the composition and function of the MSC secretome. Senescent MSCs develop a senescence-associated secretory phenotype (SASP), characterized by increased secretion of proinflammatory cytokines (e.g., IL-6, IL-8), chemokines, growth factors, and proteases, which can reinforce senescence and contribute to chronic tissue inflammation [25]. For oral MSCs, studies indicate that IL-6 and CXCL8 expression increases with donor age, suggesting an enhanced proinflammatory profile in older individuals [48]. This age-associated modulation of the secretome may have implications for regenerative therapies in older adults, highlighting the need to better understand how SASP components contribute to tissue deterioration or protection [58].

### 4.3. Tissue-of-Origin Effect on Senescence

The biological properties of MSC derived from oral tissues are influenced by both the tissue of origin and the age of the donor, generating differences in their therapeutic potential. GMSCs exhibit remarkable resistance to aging. Many of their properties, such as early proliferation (up to passage 11), expression of mesenchymal markers, and immunoregulatory capacity, are maintained even in elderly donors, up to 80 years of age according to the cohort reported by Dave et al. [13]. However, some functions, including osteogenesis and adipogenesis, decline with age, and senescent cell populations tend to increase in late in vitro passages [13]. In contrast, PDLSCs and DPSCs show greater sensitivity to age, with progressive decline in proliferation, viability, and differentiation capacity, along with an increase in senescence markers and the senescence-associated secretory phenotype (SASP) [28,35,36,37,39,43,45,48]. In comparative studies, young DPSCs have been observed to have a greater capacity for differentiation and proliferation than those from adult or elderly donors [28,36,39], while aged PDLSCs exhibit telomere shortening and attenuated proliferation, migration, and differentiation properties of the mesodermal lineage [37,48]. For this reason, DPSCs and PDLSCs from young donors are preferentially recommended for applications in osteointegration or bone regeneration, whereas GMSCs, due to their robustness against aging, may still be useful in immunomodulatory therapies even when derived from older donors. It is important to note that most of the available scientific evidence corresponds to DPSCs and PDLSCs, as they were the first dental stem cells to be isolated and characterized. Therefore, further studies are needed to systematically compare senescence-related properties according to tissue origin.

### 4.4. Donor Age

The regenerative potential of MSCs, in addition to depending on their tissue of origin, exhibits significant variations associated with donor age. These differences may be explained by the cellular senescence process that occurs with aging, as a statistically significant increase in the expression of senescence markers has been observed with advancing age [35,43,45,46].

The age groups analyzed vary among studies. Four studies compared the morphofunctional characteristics of MSCs derived from the dental pulp of deciduous teeth (SHED) and permanent teeth (DPSC) [38,40,42,50]. For deciduous teeth, participants ranged from 3 to 12 years of age. One study compared SHED with DPSC obtained from a broader population ranging from 19 to 52 years. However, Wu et al. (2015) and Yang et al. (2021) further subdivided the DPSC samples into multiple age categories, including young individuals, adults, and older adults [42,50]. This stratification enabled not only the comparison between SHED and DPSC but also age-based analyses within the DPSC group. Most studies included at least three age categories, typically encompassing adolescents, middle-aged adults, and older adults [13,35,36,37,41,42,43,45,46,48,50].

In general, DPSCs and PDLSCs derived from young donors (≤20 years) exhibit the highest proliferative and differentiation capacities, with strong osteogenic and adipogenic efficiency [36,37,39]. During early and middle adulthood (35–55 years), a gradual decline in key cellular functions has been observed, including telomere shortening, decreased proliferative capacity, extended population doubling time, and the emergence of a senescence-associated secretory phenotype (SASP). Osteogenic potential is also reduced during this stage. Although these properties tend to decline, cells may retain some biological usefulness during the initial passages in vitro [37,39,42,43,45,48]. Notably, in some studies, the reduction in proliferative properties during early and middle adulthood was mild or did not reach statistical significance compared with donors under 20 years of age [37,42].

From approximately 55 years of age onward, most DPSCs and PDLSCs populations display a marked deterioration of their morphofunctional properties. These cells exhibit a clear transition toward a senescent phenotype, with overexpression of p16^INK4A^/CDKN2A and p53, along with increased levels of proinflammatory cytokines such as IL-6 and CXCL8/IL-8. Moreover, there is a significant reduction in proliferation rate, an increase in population doubling time, and a decrease in migratory and immunomodulatory capacities, accompanied by a higher proportion of apoptotic cells [36,37,39,42,43,45,47,48]. This profile is associated with a substantial decline in regenerative potential and an enhanced secretion of proinflammatory factors [48]. Nevertheless, GMSCs appear to exhibit a relative resistance to aging, maintaining their immunomodulatory properties and proliferative even in older individuals [13]. This feature positions them as a promising cell source for anti-inflammatory, immunoregulatory, and regenerative therapies in elderly donors.

### 4.5. Cell Morphology

Cellular senescence in oral cavity DPSCs, PDLSCs, GMSCs, and PCs manifested as a fibroblastoid or fusiform phenotype while maintaining the ability to adhere to plastic, with similar characteristics observed across different age groups. However, cells from older donors exhibited increased cell size [43,45], greater cellular granularity [46], higher expression of senescence-specific markers, and more pronounced telomere shortening [13,35,43,45,46,48,49,50]. Morphologically, these senescent cells are typically flattened and enlarged, with condensed nuclei and granular cytoplasm [29,30].

The mechanisms underlying senescence-associated morphological changes have been reported to depend on the status of the scaffolding protein caveolin-1 (CAV-1), which may regulate actin stress fiber formation and focal adhesion kinase activity in senescent cells. Caveolin-1 is a structural protein component of caveolae, invaginations of the plasma membrane involved in a variety of cellular processes, including signal transduction. Growing evidence over the past 10–15 years has demonstrated a central role for caveolin-1 in the development of a senescent phenotype [60]. Lossdörfer et al. (2010) showed that the transcript expression of the senescence-associated gene caveolin-1 markedly increased with age [35]. Hence, CAV-1 has been proposed as a master regulator in the scenario of cellular senescence [29,60].

### 4.6. MSC Markers

Among the criteria for defining MSCs proposed by the International Society for Cell & Gene Therapy (ISCT) is the specific expression of surface antigens. Specifically, the MSC population must express CD73, CD90, and CD105, as measured by flow cytometry, and must lack expression of CD45, CD34, CD14, or CD11b, CD79α, or CD19, and the human leukocyte antigen (HLA) isotype [61]. According to the results obtained in this review, the classical MSC surface markers (CD73, CD90, CD105) remain stably expressed and show no association with aging. However, some non-core markers, including STRO-1, CD106, CD146, and SSEA4, show reduced expression during age-related senescence, highlighting that not all markers are equally preserved with donor age [37,43,47,62].

CD106 expression has been shown to decrease significantly in differentiated MSCs, suggesting its potential role as a marker for identifying undifferentiated MSCs [62]. Additionally, CD146+ MSCs exhibit enhanced migratory potential toward degenerated tissues; therefore, decreased CD146 expression may be associated with impaired migratory ability in senescent MSCs [29]. Furthermore, SSEA4 expression in human periodontal ligament stem cells (PDLSCs) decreases with increasing age, which may correlate with reduced differentiation capacity of the mesodermal lineage [48].

Despite the aforementioned cell surface markers indicative of senescent MSC populations, no consensus has yet been reached. Robust experimental evidence is urgently needed to identify gold-standard markers for senescent MSCs [29].

### 4.7. Molecular and Functional Markers of Senescence

Cellular senescence in MSCs encompasses molecular and functional alterations that progressively impair regenerative potential with age. Downregulation of the Wnt/β-catenin pathway promotes senescence, whereas its activation delays aging [38]. In young donors (≤20 years), Ki67 proliferation levels are higher, and PTK2 expression, linked to cell migration, is maintained, compared with older donors (>40 years), indicating diminished proliferative and migratory capacity with age. Telomere length similarly declines in older DPSCs and PDLSCs, correlating with reduced SSEA4-positive cell populations and lower proliferation [48]. Nuclear SA-β-Gal activity and p16^INK4a expression are markedly elevated in aged tissues, further confirming the senescent phenotype [50]. Epigenetic alterations, including p16 hypomethylation and decreased serine metabolism in dental pulp-derived stem cells (A-DPSCs), reinforce this phenotype by upregulating p16 expression. Together, these interrelated markers define a senescent profile in oral MSCs, underpinning their functional decline with aging.

### 4.8. Proliferation and Viability

Irreversible arrest of cell proliferation was first described in 1961 by Hayflick and Moorhead, who demonstrated that primary human cells have a limited capacity to divide due to a stable cell cycle arrest that restricts their proliferative potential [29,63]. This process, defined as cellular senescence, is induced by various endogenous and exogenous factors and is closely associated with aging [64].

Decreased proliferative capacity in senescent MSCs has been widely reported both in vitro and in vivo [29]. Indeed, cellular senescence was initially identified as a loss of proliferative capacity following extended culture, a phenomenon known as replicative senescence [29]. However, age-related senescence has also been shown to significantly reduce cell proliferation [13,37,43,45].

The colony-forming unit fibroblast (CFU-F) assay is one of the most widely used qualitative methods to estimate and evaluate the proliferation potential of MSCs in vitro [29]. Although DPSCs, PDLSCs, and GMSCs from all age groups retain the ability to form colonies, those derived from older donors exhibit reduced CFU-F levels and, significantly, form smaller colonies [13,37,43,45]. These findings suggest that harvesting MSCs from older donors may require larger tissue samples or pretreatment strategies to enhance cell proliferation and expansion.

The results analyzed indicate that the proliferative and viability capacity of DPSCs and PDLSCs is influenced by donor age [36,37,40,43,45,47,48,50]. This pattern is further supported by gene expression data, showing that genes related to mitosis and cell division are more highly expressed in cells from young donors than those from adults [40]. However, some studies report no significant differences in proliferation rate [13,42] or cell doubling time [41] at early passages. In contrast, at late passages, MSCs from adult donors progressively cease proliferation [46], suggesting accelerated deterioration associated with cellular aging during prolonged in vitro culture. Together, these findings confirm that aging negatively impacts the proliferation of dental stem cells, especially at advanced stages of in vitro culture.

Therefore, suggesting that cellular aging may be, at least partially, modulated by the in vitro microenvironment in the first passages. This observation raises the possibility that refining culture and maintenance methodologies according to donor age could help preserve or enhance the regenerative potential of aged MSCs. Adjustments such as culturing under hypoxic conditions, supplementing media with growth factors, or utilizing extracellular matrices derived from young donors may provide cues that delay senescence onset and support cellular functionality [65,66]. Developing age-adapted culture protocols could therefore represent a valuable strategy to optimize the therapeutic use of oral MSCs obtained from elderly individuals.

### 4.9. Immunomodulation

MSCs regulate both the adaptive and innate immune systems by suppressing T cell and dendritic cell maturation, reducing B cell activation and proliferation, inhibiting natural killer (NK) cell proliferation and cytotoxicity, and promoting the generation of regulatory T cells through soluble factors or cell–cell contact mechanisms [67,68].

Senescent cells secrete a variety of signaling molecules collectively known as the senescence-associated secretory phenotype (SASP). These include proinflammatory cytokines (IL-1, IL-6, IL-8), growth factors (EGF, FGF, IGF-1, PDGF, TGF-β), and other proteins that influence the tissue environment. SASP factors play key roles in inflammation, tissue remodeling, and the progression of aging and age-related diseases [29]. Although the specific components of SASP vary depending on cell type and senescence inducers, IL-6 and IL-8 are considered universal markers of SASP. Ng et al. (2020) [48] demonstrated that IL-6 and IL-8 expression were significantly higher in subjects over 20 years old. These findings align with those of Li et al. (2020) [47], who showed that immunosuppressive capacity was diminished in adult subjects. This difference arises because MSCs possess potent anti-inflammatory functions. In contrast, senescent MSCs assume a proinflammatory role due to SASP, which is considered a major contributor to the detrimental effects of aged MSCs [29].

Despite these findings, GMSCs, regardless of donor age, displayed effective immunoregulation and optimal regenerative potential in a mouse model of acute lung injury. Previous studies have shown that gingival tissues are constantly exposed to bacterial stress, leading to gingival inflammation [13,69]. Consequently, gingival mesenchymal stem cells (GMSCs) exhibit higher levels of growth factor receptors, supporting enhanced proliferation and maintenance of tissue homeostasis and secretion of anti-inflammatory cytokines [13,69]. The findings of this review are consistent with these reports, demonstrating that increased receptor expression enables GMSCs to respond effectively to growth and stress signals, thereby maintaining proliferation and homeostasis in an inflammatory or aging environment. Therefore, GMSCs represent a promising option for cell-based immunomodulatory therapeutic approaches [13].

### 4.10. Multilineage Differentiation In Vitro

The multilineage differentiation capacity of MSCs derived from the oral cavity is influenced by donor age. MSC functionality has been found to decline with age due to age-related alterations in the extracellular matrix (ECM), which further contribute to the impairment of MSC function [29]. Specifically, osteogenic and chondrogenic capacities consistently declined with age, whereas adipogenic potential showed mixed findings, with some studies reporting reduced adipogenesis and others, such as Yang et al. (2021), reporting increased adipogenic activity [47]. Despite this reduction, oral MSCs from older donors retain a degree of plasticity, particularly at early passages in vitro, suggesting they may still be viable for therapeutic applications, although with less efficiency than those from younger donors.

#### 4.10.1. Osteogenic Differentiation

There is a clear consensus among many authors regarding the decline in the osteogenic potential of MSCs with aging [13,37,42,43,44,45,46,47,50]. Several studies have demonstrated that both DPSCs and PDLSCs progressively lose their capacity for osteogenic differentiation as donor age increases [37,42,46,47,50].

This decline is manifested not only by reduced mineralization of cultures, as reported by Dave (2022) [13] and Du (2017) [45], but also by decreased expression of key osteogenic genes such as ALP, collagen type II (Col-2), Runx-2, and OPN [43,44,45].

Previous studies, including Zhu et al. [70], have described a decline in osteogenic potential beginning from middle age onwards. Overall, most reports align with the findings of the present study, describing a progressive reduction in osteogenic potential with donor age, regardless of the origin of the cells [71].

#### 4.10.2. Adipogenic Differentiation

Regarding adipogenesis, the data remain contradictory. In oral cavity MSCs, Iezzi et al. [46] and Li et al. [47] suggest a decline in adipogenic potential with age in pulp and periodontal ligament cells, respectively, while Wu et al. [43] found no significant differences between age groups. Conversely, Yang (2021) [50] reports an increase in adipogenic activity in aged DPSCs, which may be related to aging-induced changes in gene expression governing cellular differentiation. This is supported by Yang’s observation of increased expression of adipogenic genes and decreased expression of osteogenic genes in aged cells.

Numerous molecules and signaling pathways have been implicated in regulating senescent MSC lineage differentiation [50]. Peroxisome proliferator-activated receptor gamma (PPAR-γ) is considered an adipogenesis-specific transcription factor; its upregulation shifts MSC fate toward adipogenesis. Conversely, Wnt/β-catenin (WNT) signaling can restrict adipogenesis and promote differentiation toward osteoblasts [29].

Similarly to findings in other tissues, Baker et al. [72] reported that the adipogenic potential of bone marrow-derived MSCs (BMSCs) increases with age, while Choudhery et al. [71] found no change in adipogenic potential of MSCs derived from adipose tissue in elderly individuals. These discrepancies suggest that the regulation of adipogenic differentiation in MSCs may be influenced by multiple factors as yet not fully understood.

#### 4.10.3. Chondrogenic Differentiation

Regarding changes in chondrogenesis, available studies are limited, but evidence suggests a trend similar to that observed in osteogenesis—a negative correlation between donor age and the chondrogenic potential of MSCs. While investigations by Iezzi et al. [46] and Kellner et al. [39] did not find significant differences between age groups, Li et al. [47] reported an overall decline in the differentiation capacity of adult PDLSCs, including the chondrogenic lineage.

Similar findings have been reported in MSCs derived from other tissues. Choudhery et al. [71] documented a marked decrease in chondrogenic capacity associated with aging, accompanied by a significant reduction in mRNA expression of key genes such as aggrecan and collagen type II in older individuals. Similarly, Murphy et al. [73] reported a progressive loss of chondrogenic potential in MSCs with advancing age, consistent with the findings here. Collectively, this body of evidence reinforces the notion that donor age is a limiting factor in the therapeutic application of MSCs.

#### 4.10.4. Neurogenic Differentiation

A distinctive feature of MSCs is their remarkable plasticity, reflected in their ability to transdifferentiate into cell lineages beyond their mesodermal origin, including endodermal and ectodermal lineages such as neurogenic cells [74]. This transdifferentiation potential has been reported in DPSCs [36,38,42] and gingiva-derived stem cells [13].

Unlike the osteogenic lineage, the impact of aging on neurogenic differentiation appears to remain stable or decrease only slightly with age. Dave et al. [13] and Ceccarelli et al. [41] reported no significant alterations in the neurogenic differentiation capacity of MSCs derived from gingiva and periosteum with aging. However, Wu et al. [42] and Feng et al. [38] observed that aged DPSCs may lose this capacity, as evidenced by lower expression of neuronal markers in adult DPSCs compared to stem cells derived from deciduous teeth, suggesting a possible neurogenic advantage of deciduous tooth cells [38].

Previous studies have indicated that MSCs could represent a promising therapeutic option for various neurodegenerative disorders. In a recent study, Li et al. [47] showed that, although cell growth was more prominent in cultures from young donors, no significant differences were found in the total number of cells exhibiting neuronal morphology between different age groups. Similarly, no significant differences were observed in the expression of neuron-specific genes among the groups [47]. Therefore, these findings suggest that the use of MSCs for neurological applications may remain viable regardless of donor age.

### 4.11. Experimental Medicine

Although several experimental and clinical studies have investigated the use of MSCs derived from oral tissues, most have focused on young or middle-aged adults [75,76]. Evidence regarding the use of oral cavity–derived MSCs from elderly donors in experimental medicine remains very limited. A quasi-experimental study using DPSCs in adults aged 55 to 64 years reported positive effects on periodontal bone regeneration, associated with a reduction in proinflammatory interleukins [77]. However, bone regeneration with MSCs in older adults has been shown to be less effective than in younger subjects. Phase I–II studies have indicated that age affects the efficiency of bone regeneration, as the total number of cells obtained from older patients is lower and requires longer culture times compared to samples from younger individuals [78]. Therefore, despite these encouraging results, human clinical evidence supporting the use of oral cavity–derived MSCs remains limited [75], and current findings should be interpreted with caution when extrapolating their regenerative potential to older populations.

### 4.12. Future Prospects

Since the majority of studies addressing age-related differences in MSCs derived from the oral cavity have been performed in vitro, additional translational and clinical investigations are needed to confirm these findings and to evaluate the therapeutic efficacy and safety of such strategies in elderly individuals.

Furthermore, innovative strategies to rejuvenate or enhance the regenerative potential of oral MSCs should be explored, including epigenetic modulation, senolytic agents, antioxidants, autophagy regulation, microRNA treatments, preconditioning, and genetic modifications [65]. Recent studies have also demonstrated that culturing aged MSCs on extracellular matrices derived from young donors can partially restore their proliferative and differentiation potential, suggesting that a young microenvironment provides biochemical and mechanical cues capable of reversing some senescence-associated alterations [62]. These approaches could further expand the potential use of MSCs from elderly donors in regenerative applications. Overall, rejuvenating senescent MSCs represents a promising strategy to improve the efficacy of autologous MSC-based therapies, particularly in elderly patients.

### 4.13. Limitations

This review has limitations that should be acknowledged. The literature search was restricted to studies comparing MSCs from donors of different age groups, which may introduce a publication bias, as studies that found no age-related differences might remain unpublished. In addition, most studies were conducted in vitro, may not fully capture the complex in vivo behavior of MSCs. Sample sizes were often small, and there was considerable variability in experimental methods, including differences in isolation protocols, culture conditions, and differentiation assays. Furthermore, many of the included studies report only descriptive data on age-related morphological changes and do not provide standardized quantitative parameters (e.g., cell size, granularity) for assigning MSCs to specific age-related groups. It should also be noted that studies did not include sex-disaggregated analyses, preventing the evaluation of potential differences related to the donor’s sex, particularly between women of childbearing age and postmenopausal women. Recognizing these limitations provides transparency and highlights areas for improvement in future research.

## 5. Conclusions

Older age groups have become a relevant population for MSC-based therapies aimed at promoting tissue regeneration and improving function during aging. Among MSCs derived from oral cavity tissues, those from dental pulp and periodontal ligament are the most extensively studied concerning senescence-related properties. It has been demonstrated that the regenerative potential of MSCs declines with donor age, mainly after 35 years, acquiring senescence characteristics and the emergence of a proinflammatory secretory profile. These features include morphological alterations, decreased viability, reduced proliferation rates, and diminished colony-forming capacity. Moreover, aged oral MSCs show a significant decline in their differentiation potential, particularly toward osteogenic and chondrogenic lineages. In contrast, adipogenic potential tends to be maintained or even enhanced with age, while neurogenic potential may be preserved in certain cases, depending on the cell type and culture conditions.

While classical MSC surface markers (CD73, CD90, CD105) remain stably expressed and show no association with aging, some non-core markers, such as STRO-1, CD106, CD146, and SSEA4, exhibit reduced expression during age-related senescence. Moreover, some studies report no significant differences in proliferation rate or cell doubling time at early passages, and MSCs retain a degree of plasticity at these stages. Therefore, oral MSCs from elderly donors remain a promising therapeutic source. Further studies are required to explore innovative strategies aimed at rejuvenating or enhancing the regenerative potential of oral MSCs, such as hypoxic preconditioning, epigenetic modulation, or the use of senolytic agents. Integrating these approaches within the framework of personalized and precision medicine could pave the way for tailored regenerative therapies in dentistry, ultimately addressing the specific biological context of each patient and improving clinical outcomes in aging populations.

## Figures and Tables

**Table 1:** The details of the scoping review.

Scoping review title	The Effect of Aging throughout the life cycle on the Morphofunctional Characteristics of Oral Cavity Mesenchymal Stromal Cells: A Scoping Review
Review objective	To analyze the effect of age-related senescence on the morphofunctional characteristics of oral cavity MSCs.
Review question	What morphofunctional characteristics of oral cavity MSCs are altered by age-related senescence?
PICOR	P (Population): Mesenchymal stromal cells (MSCs) derived from the oral cavity I (Intervention/Issue): age-related senescence C (Comparison): MSCs obtained from young individuals O (Outcome): Morphological and functional changes R (Research design): In vitro studies

**Table 2:** Studies analyzing morphofunctional changes associated with age-related senescence.

Article	Country	Tissue of Origin	Cells	Study Groups	Cell Culture and Maintenance Methodology		
					Culture	Cell Maintenance	Experimentation Passages
Lossdörfer, S., 2010 [35]	Germany	Premolars	PDLSCs	Group 1: 12–14 years Group 2: 20–40 years Group 3: 60–75 years	Human periodontal ligament cells were scraped from the middle third of the roots.	The tissue was maintained in Dulbecco’s modified Eagle medium (DMEM) containing 10% fetal bovine serum (FBS) and 0.5% antibiotics (5000 U/mL penicillin and 5000 U/mL streptomycin) at 37 °C in an atmosphere of 100% humidity and 5% CO_2_ in air.	P5
Bressan, E., 2012 [36]	Italy	Molars	DPSCs	Group: 16–25 years Group: 26–35 years Group: 36–45 years Group: 46–55 years Group: 56–65 years Group: >66 years	The pulp was removed and immersed for 1 h at 37 °C in a digestive solution of 100 U/mL penicillin, 100 mg/mL streptomycin, 0.6 mL of 500 mg/mL clarithromycin, 3 mg/mL collagen type I, and 4 mg/mL dispase in 4 mL of 1 M PBS. Once digested, the solution was filtered through 70 mm Falcon strainers.	Non-hematopoietic (NH) stem cell expansion medium	P2–P8
Zhang, J., 2012 [37]	China	Impacted third molars	PDLSCs	Group A: 16–30 years Group B: 31–40 years Group C: 41–55 years Group D: 56–75 years	The teeth were rinsed, and the PDL was removed from the middle third of the root surface. The PDL was washed and then cut into 1 mm^3^ cubes. They were then digested with collagenase type I and dispase in α-MEM for 15 min at 37 °C with shaking.	Tissue explants were placed in culture dishes with Alpha minimum essential medium (α-MEM), 10% FBS, penicillin, streptomycin, and ascorbic acid and incubated at 37 °C with 5% CO_2_.	P3–P5
Feng, X., 2013 [38]	China	Incisors Third molars	SHEDs DPSCs	Group A: 5–12 years Group B: 45–50 years	The pulp was washed three times with PBS. The pulp tissue was then cut into pieces and placed in a solution of 3.0 mg/mL collagenase type I and 4.0 mg/mL dispase. The tissue was digested at 37 °C for 1 h and centrifuged at 1200 rpm for 8 min. Cell suspensions were seeded in 10 cm culture dishes.	The tissue was maintained in DMEM containing 10% FBS, 100 U/mL penicillin, and 100 U/mL streptomycin in a humidified atmosphere at 37 °C with 5% CO_2_. The culture medium was changed every 3 days.	P3–P5
Kellner M, 2014 [39]	Germany	Third molars	DPSCs	Group A: >22 years Group B: <22 years	The pulp was digested in 4 mg/mL collagenase type I and 2 mg/mL dispase for 1 h at 37 °C.	The cell suspension was cultured in α-MEM plus 10% FBS, 100 U/mL penicillin, and 100 μg/mL streptomycin at 37 °C and 5% CO_2_.	P1–P4
Kaukua N, 2015 [40]	Sweden		DPSCs SHEDs	Group A: 3–12 years Group B: 19–52 years	Pulp tissue was digested with 3 mg/mL collagenase I and 4 mg/mL dispase for one hour at 37 °C. Dissociated cells were passed through a 70 μm cell strainer.	The tissue was maintained in α-MEM, 10% FBS, and penicillin-streptomycin.	P1–P3
Ceccarelli G., 2015 [41]	Italy	Periosteum of the upper vestibule, lower vestibule, and hard palate	PCs	Group A: 20–30 years Group B: 40–50 years Group C: 50–60 years	Periosteum samples were digested in a solution of collagenase I (3 mg/mL) and dispase (4 mg/mL) for 1 h at 37 °C. The cells obtained from the digestion were filtered through a 70 μm strainer.	α-MEM medium supplemented with 20% FBS, 100 μM 2 p ascorbic acid, 2 mM l-glutamine, 100 U/mL penicillin, and 1000 mg/mL streptomycin at 37 °C at 5% of CO_2_. The medium was changed twice a week.	Not reported
Wu, W., 2015 [42]	China	Deciduous and permanent teeth	DPSCs SHEDs	Group A: 4 to 8 years Group B: 12 to 20 years old Group C: 30 to 50 years Group D: 55 to 67 years	The pulp was gently separated from the crown and root. It was rinsed with PBS, sectioned into 1 mm^2^ pieces, and implanted in culture dishes.	Culture medium supplemented with penicillin and streptomycin. Cells were incubated at 37 °C in a humidified atmosphere containing 5% CO_2_ and 80% humidity.	Not reported
Wu, R.X., 2015 [43]	China	Third molars	PDLSCs	Group A: 18–30 years Group B: 31–45 years Group C: 46–62 years	The teeth were rinsed with PBS, and the PDL was scraped from the middle of the root of each tooth. The PDL was cut into small blocks and digested in 3 mg/mL of collagenase type I and 4 mg/mL of dispase for 1 h. The digestive solution was then discarded and maintained in complete culture medium.	The tissues were suspended in α-MEM supplemented with 10% FBS, 0.292 mg/mL glutamine, 100 U/mL penicillin, and 100 mg/mL streptomycin, and incubated at 37 °C in a humidified atmosphere containing 5% CO_2_. The medium was changed every 3 days.	P1–P3
Yi, Q., 2016 [44]	China	Healthy teeth	DPSCs	Young people: 12–25 years Older people: 60–70 years	Dental pulp was digested in a solution of 3 mg/mL collagenase type I and 4 mg/mL dispase for 40 min at 37 °C. Single cell suspensions were obtained by passing the cells through a 70 μm strainer.	DPSCs were cultured in a humidified incubator at 37 °C with 5% CO_2_ in α-MEM supplemented with 15% FBS, 2 μM L-glutamine, 100 U/mL penicillin, and 100 U/mL streptomycin. The culture medium was changed every 3 days.	P3–P5
Du, T., 2017 [45]	China	Premolar Third molars	PDLSCs	Group A: 18–20 years (average age 19 years) Group B: 30–35 years (average age 32.5 years) Group C: 45–50 years (average age 47.5 years)	The teeth were rinsed repeatedly with PBS. PDL was scraped from the mid-root using surgical blades. The PDL tissue was minced into 1 mm^3^ cubes and then digested with collagenase type I solution in an incubator for 15 min. After centrifugation of the tissues (800 rpm), the supernatant fraction was discarded, and the tissues were resuspended and transferred to a six-well culture dish.	The tissue was maintained in α-MEM supplemented with 10% FBS and 100 U/mL penicillin-streptomycin and subsequently cultured in a humidified incubator at 37 °C with 5% CO_2_. The culture medium was changed every 3–4 days.	P2–P3
Iezzi, I., 2019 [46]	Italy	Third molars	DPSCs	Group A: 20–23 years (average age: 21 years) Group B: 42–45 years (average age: 43 years) Group C: 62–66 years (average age: 64 years)	Enzymatic isolation (3.0 mg/mL collagenase type I and 4.0 mg/mL dispase) for 1 h at 37 °C, then filtered through 70 μm cell strainers to obtain the DPSC suspension.	The tissue was maintained in DMEM/F12 supplemented with 10% FBS and 1% penicillin/streptomycin and cultured at 37 °C in a humidified atmosphere with 5% CO_2_.	Not reported
Li, X., 2020 [47]	China	Third molars	PDLSCs	Youth (YPDLSC): 19–20 years Adults (APDLSC): 35–50 years	The periodontal ligament was scraped from the middle third of the root surface and seeded in cell culture flasks.	The tissue was maintained in α-MEM supplemented with 10% FBS at 37 °C in a 5% CO_2_ atmosphere. The medium was refreshed every 2 to 3 days.	P3–P4
Ng, T.K., 2020 [48]	China	Permanent teeth	PDLSCs	Group A: ≤20 years Group B: 20–40 years Group C: >40 years	PDL was mechanically scraped from the root surface and minced, followed by digestion with 0.1% collagenase types I and III in DMEM/F12 medium containing 0.5% FBS and antibiotics for 4 to 6 h with agitation (100 rpm) at 37 °C. The cells were cultured after passing through a 40 μm cell strainer.	The tissue was maintained in high-glucose DMEM supplemented with 10% heat-inactivated FBS and 1× penicillin-streptomycin. The culture medium was changed every 3 days.	P3–P5
Sato, M., 2020 [49]	Japan	-	DPSCs	Young people: 16–18 years Senior citizens: 41–54 years	The dental pulp was minced into small pieces, followed by enzymatic digestion using 3 mg/mL type I collagenase for 45 min at 37 °C. Cells were obtained by passing the suspension through a 70 µm cell strainer.	The tissue was maintained in α-MEM supplemented with 15% FBS and 1% penicillin/streptomycin. The isolated cells were seeded at a density of 1 × 10^5^ cells per 100 mm dish.	P3–P4
Yang, R.L., 2021 [50]	China	Incisors and premolars	DPSCs SHEDs	Group A: 8 to 10 years (mean age 8.6 years) Group B: 18 to 21 years (mean age 19.4 years) Group C: 55 to 61 years (mean age 57.6 years)	DPSCs were isolated from healthy donors and cultured in complete medium.	The tissue was maintained in α-MEM supplemented with 15% FBS, 100 μg/mL glutamine, 100 μg/mL penicillin, and 100 μg/mL streptomycin, under a 5% CO_2_ atmosphere at 37 °C.	P5–P8
Dave, J.R., 2022 [13]	India	Gingival tissue during the gingivectomy or the extraction procedure	GMSCs	Group A: 13 to 31 years; mean ± SD age, 23.29 ± 6.26 years (n = 14) Group B: 37 to 55 years; mean ± SD age, 45.69 ± 6.49 years (n = 13) Group C: 59 to 80 years; mean ± SD age, 65.36 ± 6.57 years (n = 14)	The biopsy (2 × 1 × 1 mm^3^) of attached gingiva was washed in PBS. After removing the epithelial layer, it was minced into small pieces and incubated in a medium containing 0.1% collagenase and 0.2% dispase for 15 min at 37 °C. The first cell fraction was discarded, and the tissue was subjected to enzymatic digestion again for 5, 10, and 15 min. The cells were then washed and resuspended in complete medium.	The tissue was maintained in α-MEM supplemented with 10% FBS and incubated at 37 °C in 5% CO_2_.	P3–P12

**Table 3:** Morphofunctional Characteristics Reported in the Included Studies.

Article	Cell Morphology	MSC Markers	Senescence	Colony Formation and Growth Kinetics	Proliferation	PCR	Migration	Immunomodulation	In Vitro Differentiation
Lossdörfer, S., 2010 [35]	Not evaluated	Not evaluated	The transcript expression of the senescence-associated gene caveolin-1 increased markedly with age.	Not evaluated	Not evaluated	Significantly lower expression of osteoblastic marker genes in aged cultures.	Not evaluated	Not evaluated	Not evaluated
Bressan, E., 2012 [36]	Not evaluated	Not evaluated	Not evaluated	Not evaluated	Proliferative capacity decreased with increasing age. DPSCs from elderly donors show better proliferative capacity at early in vitro passages (up to passage 2).	Type II collagen was detected in the chondrogenic medium. In the osteogenic medium, collagen I, osteopontin, osteonectin, and osteocalcin were abundant. In the adipogenic medium, PPARγ, adiponectin, and GLUT4 were observed.	Not evaluated	Not evaluated	The classical adipogenic, osteogenic, and chondrogenic media were highly effective in inducing specific differentiation toward the expected cell lineages. DPSCs tend to differentiate into neuronal and bone cells rather than endothelial cells.
Zhang, J., 2012 [37]	The colonies were adherent and maintained their spindle-shaped morphology.	Negative for CD31 and CD34, and positive for CD29, CD44, CD90, CD105, CD146, and STRO-1. Expression levels of CD146 and STRO-1 decreased with age.	Not evaluated	The number of colony-forming units decreased with age.	Population doubling time was less than 24 h in all groups, with no significant differences among the four groups. The donor’s age influenced the initial cell growth rate.	Not evaluated	The migratory activity of PDLSCs decreased with increasing donor age.	Not evaluated	PDLSCs from all groups exhibited osteogenic and adipogenic potential. The differentiation capacity significantly decreased with donor aging.
Feng, X., 2013 [38]	SHED revealed a typical fibroblast-like morphology. DPSCs revealed flat and enlarged cell shapes.	Positive for CD29 and CD105, but negative for CD31 and CD34	Not evaluated	Not evaluated	Not evaluated	Not evaluated	Not evaluated	Not evaluated	Expression levels of neuronal markers, such as βIII-tubulin, microtubule-associated protein 2 (MAP2), tyrosine hydroxylase (TH), and nestin, were lower in the DPSC group than the SHED group.
Kellner, M., 2014 [39]	Not evaluated	Positive for CD29, CD44, and CD166.	Not evaluated	Not evaluated	Cells from older patients exhibit slower growth, although the maximum division potential of DPSCs does not vary significantly with age.	Not evaluated	Not evaluated	Not evaluated	No discrepancy was detected in the differentiation potential between DPSCs isolated from young and old donors.
Kaukua, N., 2015 [40]	Not evaluated	Not evaluated	Not evaluated	Not evaluated	Genes promoting cell proliferation, mitosis, and division were highly expressed in cells from deciduous tooth pulp compared to those from permanent teeth.	Genes involved in cell cycle division and mitosis were highly expressed in cells from deciduous teeth.	Not evaluated	Not evaluated	Not evaluated
Ceccarelli, G., 2015 [41]	spindle morphology	Positive for CD73, CD90, CD105, and CD29, and negative for CD45 and CD34.	Not evaluated	Not evaluated	Population doubling time ranged between 61 and 65 h, regardless of age.	Not evaluated	Not evaluated	Not evaluated	Regardless of age, in the absence of any osteogenic induction, they commit to the osteoblastic lineage after 45 days of culture.
Wu, W., 2015 [42]	Adherent cells resembling fibroblasts.	Positive expression of CD13, CD29, CD59, and CD146, and negative expression of CD19, CD24, and CD45.	Not evaluated	Not evaluated	No differences in proliferation were observed between groups at 48 h. At 72 and 96 h, DPSCs from elderly donors showed a lower proliferation rate than the other groups.	Not evaluated	Not evaluated	Not evaluated	DPSCs from deciduous teeth of children and permanent teeth of adolescents were capable of differentiating into neuronal and osteogenic lineages. DPSCs from aged teeth were completely or partially deprived of differentiation capacity.
Wu, R.X., 2015 [43]	Typical spindle-shaped morphology; cells from donors in Groups B and C were larger.	Negative for CD31 and CD45, and positive for CD90. CD146 positivity in Groups B and C was lower than in Group A	A statistically significant increase in SA-β-gal staining was observed with increasing age.	The CFU-F from young donors was greater than that formed by cells from relatively older donors.	The upward trend of the cell growth curve for Groups B and C was much slower than that of Group A.	The relative mRNA expression of the transcription factors Nanog, Oct-4, and Sox-2 showed an age-related decrease.	Not evaluated	Not evaluated	All three groups showed potential for osteogenic differentiation, but cells from Group A appeared to accumulate more calcium deposits. No significant differences were observed between the groups in adipogenic differentiation.
Yi, Q., 2016 [44]	Not evaluated	Both negative for cell surface markers CD34, CD45, and HLA-DR, and positive for CD90, CD105, and CD146	Not evaluated	Not evaluated	DPSCs from young donors showed greater proliferation capacity and significantly shorter doubling times than those from old donors.	OPN and lipoprotein lipase expressions were higher in DPSCs from young donors after osteogenic and adipogenic induction, respectively, while other markers showed no significant differences between age groups.	Not evaluated	Not evaluated	DPSCs Isolated from Old Donors Exhibited Impaired Osteogenic and Adipogenic Differentiation Potentials Mineralization was also significantly stronger in the DPSCs from young donors than in the DPSCs from old donors.
Du, T., 2017 [45]	Presented an elongated morphology, and they showed a fibroblast-like appearance.	Not evaluated	SA-β g expression increased with donor age.	The colony-forming capacity decreased significantly with increasing donor age.	The proliferative capacity of PDLSCs decreased with increasing donor age.	The expression of genes related to osteogenesis, including ALP, Col-2, and Runx-2, gradually decreased from Group A to Group C.	Not evaluated	Not evaluated	All three groups showed significant osteogenic differentiation potential. However, Group A exhibited the greatest staining intensity and area, while cells from Group C showed the lowest intensity and the smallest staining area.
Iezzi, I., 2019 [46]	No morphological differences were detected between the groups.	Positive for CD73, CD90, and CD105, and negative for CD45, HLA-DR, and CD14.	An increase in granularity and expression of SA-β-Gal and p16^INK4a was observed in Group C. No changes in cell size were evident. Telomere length in Group A was greater than in Groups B and C.	Not evaluated	After passage 13, cells from Groups B and C began to decline, and by passage 17, the DPSCs from Group C had ceased proliferation.	qRT-PCR analysis revealed age-related changes in the expression of stemness genes.	Not evaluated	Not evaluated	Reduction in adipogenic and osteogenic potential in cells from Group C compared to Groups A and B. No changes were observed in chondrogenic potential.
Li, X., 2020 [47]	APDLSCs showed irregular morphology with bifurcated cell borders. YPDLSCs exhibited a spindle-shaped morphology, abundant cytoplasm, and clear cell edges.	CD105 was positive in both groups. STRO-1 and CD146 expression was much lower in APDLSCs compared to YPDLSCs. CD45 and CD31 were negative in both groups.	Not evaluated	Not evaluated	A CCK-8 assay showed that the proliferative activity of APDLSCs was lower than that of YPDLSCs during 7 days of cell growth.	Not evaluated	Not evaluated	APDLSCs and YPDLSCs suppress the proliferation of peripheral blood mononuclear cells (PBMCs). However, APDLSCs exhibit a weaker immunosuppressive capacity than YPDLSCs.	The osteogenic, adipogenic, and chondrogenic differentiation potential of PDLSCs in the adult group is lower than that in the young group.
Ng, T.K., 2020 [48]	Not evaluated	Not evaluated	The telomere length of human PDLSCs from donors older than 40 years was significantly 2.63 times shorter than that of donors aged 20 years or younger.	Not evaluated	The proliferative potential of human PDLSCs decreased with increasing donor age.	Not evaluated	The migratory capacity of human PDLSCs was diminished in the donor age group over 40 years.	No significant differences were observed in the expression of IDO1, IDO2, HLA-E, HLA-G, IL1B, and IL10, while IL6 and IL8 were expressed at higher levels in the 20–40 and >40-year-old groups.	The mesodermal lineage differentiation capacity of human PDLSCs decreased with increasing age. The expression of SSEA4 in human PDLSCs also declined with age. However, high proliferation levels were detected at passage 2 (P2) up to 56 years of age.
Santo, M., 2020 [49]	Typical spindle-shaped morphology	Positive for CD29, CD44, CD73, CD81, CD90, and CD105, and negative for CD14 and CD34.	Telomere length, a known marker of cellular senescence, remained stable in young and old DPSCs from P1 to P3.	Not evaluated	DPSCs from older patients showed lower initial proliferation but reached levels similar to those of younger patients from passage 3 onwards, with no significant differences throughout the entire period.	Not evaluated	Not evaluated	Not evaluated	Not evaluated
Yang, R.L., 2021 [50]	Not evaluated	Not evaluated	The level of p16 in A-DPSCs was higher than that in Y-DPSCs and SHEDs.	Not evaluated	A-DPSCs showed a lower proliferation rate than SHED and Y-DPSCs, and the latter proliferated less than SHEDs.	With increasing age, there was a decrease in the expression of odontogenic/osteogenic differentiation genes and an increase in the expression of adipogenic differentiation genes.	Not evaluated	Not evaluated	The osteogenic differentiation capacity of DPSCs decreased in an age-dependent manner. The adipogenic differentiation capacity of A-DPSCs was slightly higher than that of SHED and Y-DPSCs, indicating that adipogenic activity increased with aging.
Dave, J.R., 2022 [13]	Morphology of fibroblasts adhering to plastic	Positive for CD44, CD90, CD73 and CD105, without any contamination of hematopoietic cells (CD34 and CD45)	Group C showed a significantly larger population of senescent cells.	They formed efficient colonies; those of group A were larger than those of group C.	GMSCs from groups A, B, and C showed similar PDT at passages 9 and 11; however, PDT of group C increased significantly at passage 13.	Not evaluated	GMSCs in Group B showed a significantly higher migration rate than Groups A and C.	Not evaluated	Group A showed early signs of adipogenic differentiation compared to groups B and C. There was a significant decrease in mineralization in the osteogenic cultures of groups B and C. No decrease in the neurogenic differentiation capacity of GMSCs was observed with donor age.

## Data Availability

No new data were created or analyzed in this study. Data sharing is not applicable to this article.

## Abbreviations

The following abbreviations are used in this manuscript:

DPSCs	Dental pulp stem cells
SHEDS	Stem cells from exfoliated deciduous teeth
PDLSCs	Periodontal ligament stem cells
MSC	Mesenchymal stromal cell
FBS	Fetal bovine serum
α-MEM	Alpha minimum essential medium
DMEM	Dulbecco’s modified Eagle medium
PBS	Phosphate-buffered saline
CD	Cluster of differentiation
PDT	Population doubling time
qRT-PCR	Quantitative reverse transcription polymerase chain reaction
HLA-DR	Human leukocyte antigen—DR isotype
PBMC	Peripheral blood mononuclear cell
SA-β-gal	Senescence-associated beta-galactosidase
ALP	Alkaline Phosphatase
CFU	Colony-forming unit
PC	Periosteal cells
ISCT	International Society for Cell & Gene Therapy
CAV-1	Caveolin-1
